# Estimating causal effects of atherogenic lipid-related traits on COVID-19 susceptibility and severity using a two-sample Mendelian randomization approach

**DOI:** 10.1186/s12920-021-01127-2

**Published:** 2021-11-13

**Authors:** Masahiro Yoshikawa, Kensuke Asaba, Tomohiro Nakayama

**Affiliations:** 1 Division of Laboratory Medicine, Department of Pathology and Microbiology, Nihon University School of Medicine, Tokyo, Japan; 2 Department of Computational Diagnostic Radiology and Preventive Medicine, The University of Tokyo Hospital, Tokyo, Japan

**Keywords:** COVID-19, SARS-CoV-2, Triglyceride, LDL-cholesterol, Apolipoprotein B, Mendelian randomization

## Abstract

**Background:**

As the number of COVID-19 deaths continues to rise worldwide, the identification of risk factors for the disease is an urgent issue, and it remains controversial whether atherogenic lipid-related traits including serum apolipoprotein B, low-density lipoprotein (LDL)-cholesterol, and triglyceride levels, are risk factors. The aim of this study was to estimate causal effects of lipid-related traits on COVID-19 risk in the European population using a two-sample Mendelian randomization (MR) approach.

**Methods:**

We used summary statistics from a genome-wide association study (GWAS) that included 441,016 participants from the UK Biobank as the exposure dataset of lipid-related traits and from COVID-19 Host Genetics Initiative GWAS meta-analyses of European ancestry as the outcome dataset for COVID-19 susceptibility (32,494 cases and 1,316,207 controls), hospitalization (8316 cases and 1,549,095 controls), and severity (4792 cases and 1,054,664 controls). We performed two-sample MR analyses using the inverse variance weighted (IVW) method. As sensitivity analyses, the MR-Egger regression, weighted median, and weighted mode methods were conducted as were leave-one-out sensitivity analysis, the MR-PRESSO global test, PhenoScanner searches, and IVW multivariable MR analyses. A *P* value below 0.0055 with Bonferroni correction was considered statistically significant.

**Results:**

This MR study suggested that serum apolipoprotein B or LDL-cholesterol levels were not significantly associated with COVID-19 risk. On the other hand, we inferred that higher serum triglyceride levels were suggestively associated with higher risks of COVID-19 susceptibility (odds ratio [OR] per standard deviation increase in lifelong triglyceride levels, 1.065; 95% confidence interval [CI], 1.001–1.13; *P* = 0.045) and hospitalization (OR, 1.174; 95% CI, 1.04–1.33; *P* = 0.012), and were significantly associated with COVID-19 severity (OR, 1.274; 95% CI, 1.08–1.50; *P* = 0.004). Sensitivity and bidirectional MR analyses suggested that horizontal pleiotropy and reverse causation were unlikely.

**Conclusions:**

Our MR study indicates a causal effect of higher serum triglyceride levels on a greater risk of COVID-19 severity in the European population using the latest and largest GWAS datasets to date. However, as the underlying mechanisms remain unclear and our study might be still biased due to possible horizontal pleiotropy, further studies are warranted to validate our findings and investigate underlying mechanisms.

**Supplementary Information:**

The online version contains supplementary material available at 10.1186/s12920-021-01127-2.

## Background

The World Health Organization has reported that the number of deaths from coronavirus disease 2019 (COVID-19) caused by severe acute respiratory syndrome coronavirus 2 (SARS-CoV-2) continues to rise worldwide (over 4.6 million as of September 2021) [1]. Therefore, in addition to the establishment of effective therapies, the identification of risk factors for COVID-19 is an urgent issue. Observational studies have reported that the severity of COVID-19 depends on risk factors such as obesity, coronary artery disease (CAD), and diabetes [2, 3]. Although dyslipidemia is associated with these risk factors [4–6], different results from observational studies have been reported with regard to an association between dyslipidemia and COVID-19 risk [7–11]. Moreover, observational studies tend to suffer from bias due to possible confounders and reverse causation [12].

The Mendelian randomization (MR) method mimics a study with a randomized controlled design using single-nucleotide variants (SNVs) (also called single-nucleotide polymorphisms [SNPs]) as instrumental variables (IVs) and can estimate causal effects of risk factors on diseases of interest. According to Mendel’s law, genetic variants are randomly assigned at meiosis. Therefore, MR studies are less likely to suffer from possible confounders or reverse causation, which as stated above are limitations of observational studies [12]. A recent genome-wide association study (GWAS) using UK Biobank (UKBB) data and MR analysis [13] reported that among atherogenic lipid-related traits (apolipoprotein B [Apo-B], low-density lipoprotein cholesterol [LDL-C], and triglyceride [TG]), Apo-B accounted for the causal effect on CAD risk, independently of LDL-C and TGs. Nevertheless, the estimated effects of atherogenic lipid-related traits on COVID-19 risk are inconsistent even among MR studies [14–17]. The aim of the present study was to estimate causal effects of serum Apo-B, LDL-C, and TG levels on risk of COVID-19 susceptibility, hospitalization, and severity in the European population using a two-sample MR approach.

## Methods

### Study design

We performed two-sample univariable MR analyses using summary-level GWAS datasets to estimate causal effects of circulating atherogenic lipid-related traits on COVID-19 using genetically predicted serum Apo-B, LDL-C, and TG levels as exposures and risk of COVID-19 susceptibility, hospitalization, and severity as outcomes. To examine reverse causation, we also performed bidirectional two-sample univariable MR analyses using genetically predicted risk of COVID-19 susceptibility, hospitalization, and severity as exposures and serum Apo-B, LDL-C, and TG levels as outcomes.

All analyses were conducted using the TwoSampleMR package (version 0.5.6) in R software (version 4.0.3) [18].

A *P* value below 0.0055 (0.005/3/3 by Bonferroni correction) was considered statistically significant and a *P* value between 0.0055 and 0.05 was considered suggestively significant in the MR analyses.

### Data sources

For the exposure dataset of genetically predicted serum Apo-B, LDL-C, and TG levels, summary statistics were available from a GWAS [13] that included up to 441,016 participants from UKBB. To our knowledge, this is to date the largest GWAS in sample size of atherogenic lipid-related traits. The data were standardized and normalized such that the mean was 0 and the standard deviation (SD) was 1. However, the mean and SD of TG were not provided in the GWAS; instead, the median TG was given as 1.50 (IQR = 1.11) mmol/L. The mean (SD) TG of 470,434 participants in UKBB was 1.75 (1.02) mmol/L [19]. The GWAS datasets are publicly available from the IEU open GWAS database [20] as GWAS-IDs of “ieu-b-108” for serum Apo-B level, “ieu-b-110” for serum LDL-C level, and “ieu-b-111” for serum TG level.

For the outcome dataset of genetically predicted risk of COVID-19 susceptibility, hospitalization, and severity, summary statistics were available from the GWAS meta-analyses by COVID-19 Host Genetics Initiative (COVID-19-HGI) [21] (Round 5) of European ancestry, which excluded UKBB data. Therefore, in our two-sample MR study, possible study overlap between the exposure and outcome datasets was unlikely. The outcome dataset included 1,348,701 participants (32,494 laboratory confirmed cases of SARS-CoV-2 infection and 1,316,207 population controls) for COVID-19 susceptibility, 1,557,411 participants (8316 hospitalized COVID-19 patients and 1,549,095 population controls) for COVID-19 hospitalization, and 1,059,456 participants (4792 very severe respiratory confirmed COVID-19 cases and 1,054,664 controls) for COVID-19 severity. COVID-19-HGI defined very severe respiratory confirmed COVID-19 cases as patients hospitalized for laboratory-confirmed SARS-CoV-2 infection who died or were given respiratory support. The dataset (Round 5) was the latest version with the largest sample size involving European ancestry released on January 18, 2021 [22].

For the dataset of genetically predicted body mass index (BMI) trait, summary statistics are publicly available from a GWAS of European ancestry (a meta-analysis of GIANT [Genetic Investigation of ANthropometric Traits] consortium studies and UKBB with a total of 681,275 participants) [23], and from the IEU open GWAS database [20] as GWAS-ID of “ieu-b-40”.

### Selection of instrumental variables

MR analyses use SNVs as IVs that must satisfy the following three assumptions [24]: the IVs are associated with the exposure; the IVs affect the outcome only via exposure; and the IVs are not associated with confounders.

To estimate causal effects, we selected SNVs from the exposure GWAS dataset as IVs by clumping together all SNVs associated with the trait with *P* < 5.0 × 10^–8^ (a genome-wide significance level) and not in linkage disequilibrium with other SNVs (r^2^ < 0.001, and distance > 10,000 kb) using the clump_data function (population = "EUR"). We extracted the summary statistics for each SNV from both the exposure and outcome GWAS datasets and then harmonized them. We did not include proxy SNVs in the analysis [25, 26]. We excluded palindromic SNVs with an intermediate minor allele frequency (MAF) > 0.42 [24, 27]. To evaluate the strength of the IVs, the F-statistic for each SNV was calculated using the following formula: F-statistic = R^2^ × (N − 2)/(1 − R^2^), where R^2^ is the proportion of variance in phenotype explained by each SNV in exposure, and N is the sample size. We calculated R^2^ using the following formula: R^2^ = 2 × (Beta/SD)^2^ × (MAF) × (1 − MAF), where Beta is the per allele effect size of the association between each SNV and phenotype [28]. IVs with an F-statistic below 10 (if any existed) were considered weak instruments [29].

### Two-sample Mendelian randomization and sensitivity analyses

The Wald ratio, which estimates the causal effect of each IV exposure on outcome, was calculated as the ratio of Beta for the corresponding SNV in the outcome dataset divided by Beta for the same SNV in the exposure dataset [24]. We conducted a meta-analysis of each Wald ratio using the inverse variance weighted (IVW) method as a main analysis to estimate the overall causal effect of genetically predicted values of the exposure on the outcome. For the IVW method, we used a multiplicative random-effects model when Cochran’s Q statistic (as described below) was significant (*P* < 0.05) [30]; otherwise, a fixed-effects model was used. Based on IVW results, we inferred the causal effect of the lifelong change in exposure on the outcome [31].

In addition, we conducted sensitivity analyses using the MR-Egger regression method, the weighted median method, the weighted mode method, and leave-one-out sensitivity analysis. The MR-Egger regression method can detect horizontal pleiotropy. The MR-Egger intercept is nonzero with statistical significance (*P* < 0.05) if possible horizontal pleiotropy of IVs exists [32]. The weighted median method can generate a valid causal estimate if at least 50% of the instrument SNVs satisfy the IV assumptions [32]. The weighted mode method forms clusters of individual SNVs and estimates the causal effect from the largest cluster [32]. Leave-one-out sensitivity analysis removed each SNV from the IVW method and re-estimated the causal effect to assess the reliability of the analysis [33]. We also measured heterogeneity among causal estimates across all SNVs in the IVW method by calculating Cochran’s Q statistic and the corresponding *P* value. Low heterogeneity (*P* > 0.05) provides more reliability for causal effects [34]. Regarding MR to estimate the causal effect of TGs on COVID-19 severity, we added the following two analyses: we searched for SNVs associated with *P* < 5.0 × 10^–8^ with pleiotropic effects on BMI and any type of leukocyte or inflammatory marker using the web tool PhenoScanner (version 2) [35, 36] and then excluded them from the IVW method; we conducted the MR-PRESSO (Pleiotropy RESidual Sum and Outlier) global test with the run_mr_presso function to detect possible horizontal pleiotropy [37]. Moreover, we conducted IVW multivariable MR analyses with the mv_ivw function to estimate the direct effects of genetically predicted TG levels on risk of COVID-19 independent of the effects of other exposure traits using genetically predicted Apo-B, LDL-C, or BMI traits as a covariate [13, 38–41].

### Statistical power

We calculated statistical power in the MR analyses at a type-I error rate of 0.05 using the web tool mRnd [42, 43], as shown in Additional file 1: Table S1. For example, we achieved 80% power to detect an odds ratio (OR) of 1.066 (or 0.934) for the causal effect of genetically predicted Apo-B levels on COVID-19 susceptibility.

## Results

**Fig. 1:**
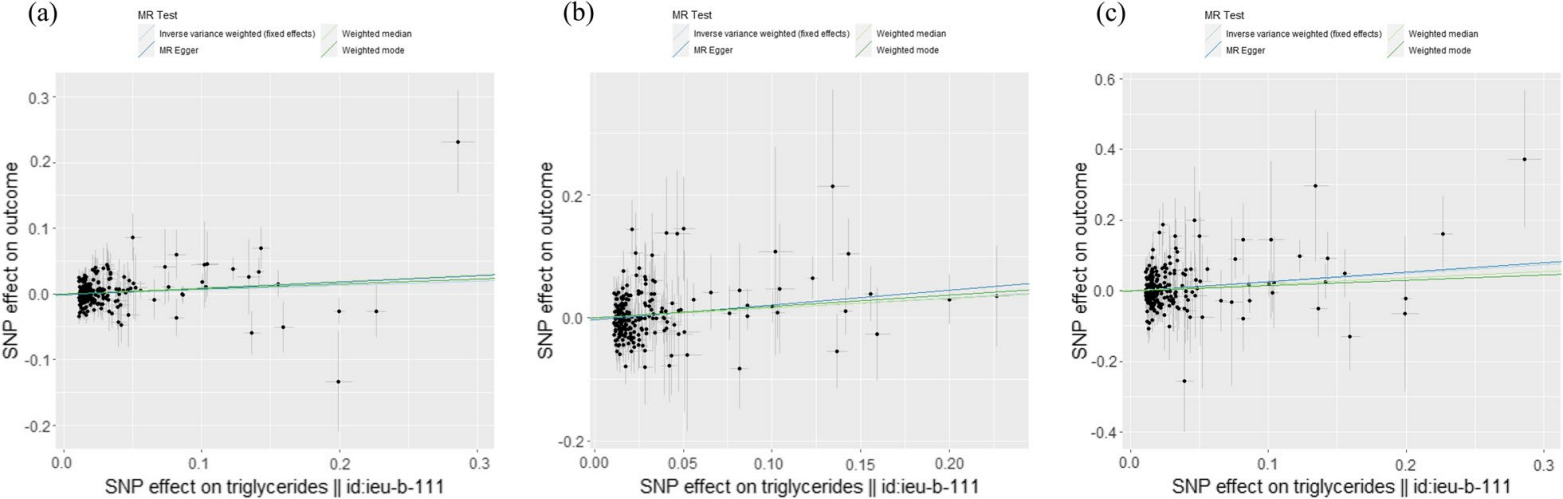
Scatter plots for estimating causal effects of genetically predicted serum TG levels on risk of **a** susceptibility, **b** hospitalization, and **c** severity. Each black point representing the effect sizes of each SNP on the exposure (horizontal-axis) and on the outcome (vertical-axis) is plotted with error bars corresponding to each standard error (SE). The slope of each line corresponds to the combined estimate using each method of the IVW (light blue line), the MR-Egger regression (blue line), the weighted median (light green line), and the weighted mode (green line)

The characteristics of all SNVs included in our univariable MR analyses are shown in Additional file 1: Tables S2–S4. The F-statistic for every instrument was > 25, indicating no weak instrument bias. During harmonization, several SNVs were excluded because they were palindromic with intermediate MAFs (“palindromic ambiguous” was “TRUE” in Additional file 1: Tables S2–S4). The overall univariable MR results are shown in Table 1, Fig. 1, and Additional file 2: Figures S1–S3. None of the MR methods, including IVW, indicated any causal effect of genetically predicted Apo-B or LDL-C levels on risk of COVID-19, whereas some MR methods did indicate causal effects of genetically predicted TG levels (Table 1). By employing the IVW methods, we inferred that lifelong elevated TG levels had suggestive causal effects on a higher risk of COVID-19 susceptibility (OR per 1-SD increase in TGs, 1.065; 95% CI, 1.001–1.13; *P* = 0.045) and hospitalization (OR, 1.174; 95% CI, 1.04–1.33; *P* = 0.012). Moreover, IVW allowed us to infer that lifelong elevated TG levels had a significant causal effect on a higher risk of COVID-19 severity (OR, 1.274; 95% CI, 1.08–1.50; *P* = 0.004). The MR-Egger intercept indicated little evidence of horizontal pleiotropy (*P* = 0.75), and Cochran’s Q statistic for the IVW method indicated low heterogeneity (*P* = 0.43) and reliability of the causal effect. Leave-one-out sensitivity analysis (Additional file 2: Figure S3a) revealed the reliability of the IVW analysis, and the MR-PRESSO global test (*P* = 0.44) suggested a lack of possible horizontal pleiotropy. Furthermore, a funnel plot (Additional file 2: Figure S3b) depicted general symmetry, suggesting little evidence of heterogeneity or horizontal pleiotropy [32]. The weighted median and weighted mode methods also showed OR scales and directions consistent with the IVW method; however, the effects were not significant, raising the possibility of horizontal pleiotropy and confounding. Therefore, we performed PhenoScanner searches to identify SNVs associated with possible pleiotropic effects on other risk factors for COVID-19 at *P* < 5.0 × 10^–8^. Among 208 SNVs used as IVs for serum TG levels, we identified 17 SNVs associated with BMI (rs10797119, rs10811662, rs10883026, rs11030107, rs13389219, rs2937124, rs2943645, rs3808477, rs3814883, rs394872, rs60856912, rs62135012, rs684773, rs7239575, rs7947951, rs921971, and rs998584), and 20 SNVs associated with any type of leukocyte or inflammatory marker (rs11187019, rs12185242, rs1292065, rs174566, rs2382825, rs28383314, rs326222, rs3775228, rs3814883, rs4665972, rs4761234, rs60856912, rs61905078, rs62427982, rs6432622, rs6999569, rs7239575, rs72603744, rs483082 [C-reactive protein (CRP)], and rs79287178 [TRAIL (tumor necrosis factor-related apoptosis inducing ligand) level]). We obtained results comparable to those of the original IVW method when we excluded the 17 SNVs or 20 SNVs (for the former: OR, 1.271; 95% CI, 1.07–1.51; *P* = 0.0059; number of SNVs, 191; for the latter: OR, 1.341; 95% CI, 1.10–1.64; *P* = 0.004; number of SNVs, 188).

**Table 1:** Univariable MR results of the effect of genetically predicted atherogenic lipid-related traits on COVID-19 risk

Exposure traits	Outcome traits	N of SNVs	IVW method	MR-Egger regression method		Weighted median method	Weighted mode method	Heterogeneity (IVW method)
			OR (95% CI) *P*-value	OR (95% CI) *P*-value	Intercept (SE) *P*-value	OR (95% CI) *P*-value	OR (95% CI) *P*-value	Cochran’s Q *P*-value
Apo-B	COVID-19 susceptibility	128	0.995 (0.93–1.07) 0.89	1.013 (0.91–1.12) 0.81	− 0.0009 (0.0019) 0.64	1.057 (0.93–1.19) 0.38	1.026 (0.93–1.14) 0.63	128.4 0.45
Apo-B	COVID-19 hospitalization	126	0.995 (0.85–1.16) 0.94	0.909 (0.72–1.15) 0.43	0.0040 (0.0041) 0.33	0.902 (0.71–1.15) 0.41	0.881 (0.68–1.15) 0.35	125.7 0.47
Apo-B	COVID-19 severity	130	1.033 (0.82–1.31) 0.79	0.933 (0.64–1.35) 0.71	0.0046 (0.0065) 0.48	0.935 (0.67–1.31) 0.70	0.979 (0.70–1.36) 0.90	172.0 0.007
LDL-C	COVID-19 susceptibility	114	1.048 (0.96–1.14) 0.27	1.028 (0.89–1.18) 0.70	0.0008 (0.0023) 0.74	1.056 (0.93–1.20) 0.41	1.025 (0.90–1.17) 0.71	114.2 0.45
LDL-C	COVID-19 hospitalization	113	0.986 (0.83–1.17) 0.86	0.853 (0.64–1.14) 0.28	0.0057 (0.0046) 0.22	0.892 (0.68–1.17) 0.41	0.889 (0.67–1.18) 0.42	108.5 0.58
LDL-C	COVID-19 severity	115	1.034 (0.83–1.29) 0.77	0.806 (0.54–1.21) 0.30	0.0099 (0.0067) 0.14	0.882 (0.62–1.26) 0.49	0.949 (0.65–1.38) 0.79	127.1 0.19
TG	COVID-19 susceptibility	208	1.065 (1.00–1.13) 0.045	1.101 (0.99–1.21) 0.052	− 0.0014 (0.0016) 0.36	1.099 (0.99–1.22) 0.07	1.079 (0.98–1.19) 0.13	235.9 0.08
TG	COVID-19 hospitalization	205	1.174 (1.04–1.33) 0.012	1.275 (1.06–1.53) 0.010	− 0.0036 (0.0030) 0.23	1.167 (0.96–1.42) 0.12	1.201 (1.01–1.43) 0.039	192.5 0.71
TG	COVID-19 severity	208	1.274 (1.08–1.50) 0.004	1.310 (1.03–1.67) 0.030	− 0.0013 (0.0040) 0.75	1.201 (0.92–1.57) 0.18	1.160 (0.91–1.47) 0.22	210.2 0.43

*Apo-B*, apolipoprotein B; *CI*, confidence interval; *COVID-19*, coronavirus disease 2019; *IVW*, inverse variance weighted; *LDL-C*, low-density lipoprotein cholesterol; *MR*, Mendelian randomization; *N*, number; *OR*, odds ratio; *SE*, standard error; *SNV*, single nucleotide variant; *TG*, triglyceride

Moreover, we carried out IVW multivariable MR analyses as sensitivity analyses to estimate the direct causal effect of genetically predicted TG levels on COVID-19 risk adjusted for each of the genetically predicted Apo-B, LDL-C, and BMI traits. The IVW multivariable MR results are shown in Table 2. The suggested causal effect of TGs on COVID-19 susceptibility was eliminated upon adjustment for each trait. We obtained comparable results regarding the causal effect of TGs on COVID-19 hospitalization and severity after adjustment for each trait; however, the latter had only suggestive significance.

**Table 2:** IVW multivariable MR for the effects of TGs on COVID-19 risk adjusted for other traits

Exposure traits	Adjustment	Outcome traits		
		COVID-19 susceptibility	COVID-19 hospitalization	COVID-19 severity
		OR (95% CI), *P*-value	OR (95% CI), *P*-value	OR (95% CI), *P*-value
Apo-B	TG	0.966 (0.89–1.05), 0.422	0.905 (0.77–1.06), 0.228	0.954 (0.73–1.24), 0.725
TG	Apo-B	1.063 (0.98–1.15), 0.139	1.178 (1.01–1.37), 0.036	1.306 (1.06–1.60), 0.011
LDL	TG	1.017 (0.93–1.12), 0.717	0.948 (0.79–1.13), 0.558	1.036 (0.79–1.35), 0.795
TG	LDL-C	1.073 (0.99–1.16), 0.073	1.158 (1.00–1.34), 0.042	1.276 (1.05–1.55), 0.013
BMI	TG	1.130 (1.02–1.25), 0.016	1.374 (1.13–1.67), 0.001	1.404 (1.08–1.83), 0.012
TG	BMI	1.071 (0.99–1.15), 0.066	1.189 (1.04–1.36), 0.013	1.261 (1.04–1.53), 0.018

*Apo-B*, apolipoprotein B; *BMI*, body mass index; *CI*, confidence interval; *COVID-19*, coronavirus disease 2019; *IVW*, inverse variance weighted; *LDL-C*, low-density lipoprotein cholesterol; *MR*, Mendelian randomization; *OR*, odds ratio; *SE*, standard error; *SNV*, single nucleotide variant; *TG*, triglyceride

Finally, we examined reverse causation by performing bidirectional two-sample univariable MR analyses using genetically predicted risks of COVID-19 as exposures and atherogenic lipid-related traits as outcomes. The results are shown in Additional file 3: Table S5. Overall, numbers of instrumental SNVs were low, and heterogeneities were high. We found that none of the COVID-19 risks had any significant causal effects on atherogenic lipid-related traits.

## Discussion

From univariable MR studies, we inferred suggestive causal effects of lifelong higher TG levels on higher risk of COVID-19 susceptibility (OR, 1.065; 95% CI, 1.001–1.13; *P* = 0.045) and hospitalization (OR, 1.174; 95% CI, 1.04–1.33; *P* = 0.012) and a significant causal effect of TGs on COVID-19 severity (OR, 1.274; 95% CI, 1.08–1.50; *P* = 0.004) but could not find any causal effect of Apo-B or LDL-C levels. The suggested effect of TGs on COVID-19 susceptibility was eliminated and the significant effect on COVID-19 severity was attenuated with suggestive significance after adjustment each for Apo-B, LDL-C, and BMI traits in IVW multivariable MR analyses. Nonetheless, our bidirectional MR study indicated that reverse causation of COVID-19 risk on atherogenic lipid-related traits was unlikely.

Observational studies have reported contradictory results. A study among 9005 UKBB participants (1508 patients testing positive for SARS-CoV-2 and 7497 controls) reported that Apo-B, LDL-C, and TGs were not significantly associated with SARS-CoV-2 infection [7]. A systematic review and meta-analysis including 23 studies involving 10,122 COVID-19 patients showed that hospitalized patients with severe disease or non-survivor status had significantly lower serum LDL-C but not TG levels compared to patients with milder disease or survivor status; however, only a few studies of those with European ancestry were included [8]. A retrospective single-center study with 654 patients in Spain showed that LDL-C < 69 mg/dl at admission was independently associated with a greater risk of 30-day mortality from COVID-19 (hazard ratio, 1.94; 95% CI, 1.14–3.31, *P* = 0.014) [9]. However, a prospective single-center study with 48 COVID-19 patients in France did not detect a significant relationship between LDL-C and 28-day mortality [10]. A retrospective single-center study with 600 COVID-19 patients in the United States reported hypertriglyceridemia as being associated with mortality (OR, 2.3; 95% CI, 1.4–3.7; *P* = 0.001) independent of obesity, high CRP, and high leukocyte count [11]. Some observational studies of European-ancestry subjects were consistent with our MR results, though others were not. Such discrepancies may be due to the small sample size and retrospective and/or single-center designs of observational studies. Moreover, observational studies tend to suffer from bias by possible confounders and reverse causation [12]. For example, serum lipid levels might be lower as a result of poor nutrition status due to COVID-19 severity [8]. Associations between serum lipid levels and COVID-19 risks might be secondary to immune-inflammatory responses that could worsen COVID-19 outcomes [8, 9, 11], though the association of LDL-C with COVID-19 mortality was independent of inflammatory markers in the above Spanish study [9]. MR studies can overcome such limitations of observational studies [12]. Regardless, estimates of MR studies tend to be larger than those of observational studies, as the former estimate lifelong rather than short-term effects [31].

Some MR studies have investigated the causal effects of atherogenic lipid-related traits on COVID-19 risk. Leong A et al. [14] did not find any causal effect of LDL-C or TGs on COVID-19 susceptibility or severity that was significant after Bonferroni correction (LDL-C and susceptibility: OR, 1.09; 95% CI, 1.01–1.18; *P* = 0.04; LDL-C and severity: OR, 1.11; 95% CI, 0.98–1.26; *P* = 0.11; TG and susceptibility: OR, 1.05; 95% CI, 0.99–1.11; *P* = 0.08; TG and severity: OR, 1.05; 95% CI, 0.95–1.15; *P* = 0.36) using Global Lipids Genetic Consortium (GLGC) GWAS data for LDL-C and TG traits for 188,577 participants of European ancestry [44] and COVID-19-HGI GWAS meta-analyses data (Round 4). Aung N et al. [15] used two-sample MR with IVW methods to investigate causal effects of LDL-C and TGs on COVID-19 susceptibility (for LDL-C: OR, 1.20; 95% CI, 1.06–1.37, *P* = 0.006; for TG: OR, 1.10; 95% CI, 0.79–1.53; *P* = 0.58) using GLGC GWAS data [44] and COVID-19-HGI GWAS meta-analyses data (Round 2). Ponsford MJ et al. [16] reported no effect of LDL-C on COVID-19 with respiratory failure (OR, 0.85; 95% CI, 0.61–1.20) using GLGC GWAS data [44] and COVID-19 GWAS data involving 1610 cases with respiratory failure and 2205 controls in Italy and Spain [45]. Recently, Zhang et al. [17] reported that Apo-B had a causal effect on COVID-19 susceptibility (OR, 1.18; 95% CI, 1.07–1.29; *P* = 0.001) using GWAS data of the Apo-B trait for 24,925 participants of European ancestry and UKBB data with 1221 COVID-19 cases and 4117 controls. However, they did not find any causal effect of Apo-B, LDL-C or TGs on COVID-19 severity (for Apo-B: OR, 0.95; 95% CI, 0.84–1.07; *P* = 0.36; for LDL-C: OR, 0.96; 95% CI, 0.85–1.09; *P* = 0.51; for TG: OR, 0.94; 95% CI, 0.78–1.15; *P* = 0.56) using LDL-C and TG traits GWAS for less than 300,000 participants of European ancestry and COVID-19 GWAS data obtained in Italy and Spain [45]. We performed our MR study using the latest GWAS datasets with the largest sample sizes to date, which might explain the discrepancies between our and previous MR studies. In a preprint article, Sun Y et al. [46] performed an MR study using the UKBB dataset for the TG trait (given as GWAS-ID of “ukb-d-30870_irnt”), COVID-19-HGI GWAS meta-analyses data, and COVID-19 GWAS data obtained in Italy and Spain [45]. Consistent with our MR study, they found that higher TGs had causal effects on the risk of severe COVID-19 (for 2972 very severe respiratory confirmed covid cases: beta, 0.219; *P* = 0.021; for 6492 hospitalized cases: beta, 0.224; *P* = 0.001; for 1610 cases with respiratory failure: beta, 0.446; *P* = 0.006).

The underlying mechanisms by which hypertriglyceridemia worsens COVID-19 outcomes clinically and pathologically remain unclear. Although an MR study showed that genetically predicted lower counts of basophils and myeloid white blood cells had causal effects on COVID-19 severity [47], an observational study found positive correlations of higher leukocyte counts and CRP with TGs in COVID-19 patients and suggested that hypertriglyceridemia might have a direct effect on COVID-19 severity due to an enhanced inflammatory response [11]. In fact, studies suggested that hypertriglyceridemia could promote inflammation through leukocyte activation [48], macrophage accumulation in several organs [49] and increased sensitivity to cytokine stimulation of aortic endothelial cells [50]. Moreover, an MR study indicated causal effects of cardiometabolic exposures, including BMI and TGs, on circulating proteins that might contribute to severe COVID-19 [51]. For example, univariable MR indicated that both BMI and TGs had causal effects on reducing immunoglobulin G (IgG), a class of antibodies that help protect against infection. Nonetheless, multivariable MR indicated that BMI indirectly lowers IgG due to its influence on raising serum TG levels [51]. Therefore, we infer that hypertriglyceridemia may worsen COVID-19 at least partly through the direct causal effect of TGs on inflammatory responses.

There are several major limitations to be noted in the present MR study. First, our MR analyses estimating a causal effect of TGs on COVID-19 severity using the weighted median and weighted mode methods had directionally consistent results but no statistical significance. Although so did other MR analyses [15, 37, 52], we must pay attention to possible horizontal pleiotropy and confounding. Therefore, we attempted to exclude possible pleiotropic effects by performing Phenoscanner searches. Previous MR studies have indicated that BMI is a risk factor for COVID-19 severity [14, 15, 37, 52], but CAD and diabetes are unlikely [14, 53]. Therefore, we excluded SNVs associated with BMI as well as inflammatory responses from the IVW method. Moreover, we conducted IVW multivariable MR analyses to eliminate the effect of Apo-B, LDL-C, or BMI traits. Regardless, we obtained results comparable to those of original IVW methods estimating causal effects of TGs on COVID-19 hospitalization and/or severity. Although the multivariable MR results had only suggestive significance, Bonferroni correction can be considered overly conservative, given the high correlation between lipid-related traits [13]. Therefore, it is suggested that hypertriglyceridemia increases risk of COVID-19 hospitalization and severity to some extent independently of the effects of BMI, inflammatory responses, and other atherogenic lipid-related traits. Second, as described in the Methods section and Additional file 1: Table S1, our MR analyses estimating the causal effects of Apo-B and LDL-C on risk of COVID-19 might not possess sufficient statistical power to detect, if any existed, a weak association. Third, an observational study found a U-shaped association between LDL-C and COVID-19 severity [54]. However, as two-sample MR analysis based on summary-level data assumes a linear relationship between exposure and outcome, we could not test for a nonlinear relationship between LDL-C and COVID-19 severity [38]. Fourth, our MR analysis was based on populations of European ancestry, and the findings are unlikely to be generalized to other populations. Fifth, the GWAS of lipid-related traits was conducted, including blood samples during a non-fasting status that might affect serum lipid or lipoprotein levels [13, 55], which may have affected our causal estimate of TGs on the risks of COVID-19. Nonetheless, the GWAS and MR study adjusted for fasting time led to negligible changes in the effect estimates of Apo-B, LDL-C, and TGs on a higher risk of CAD [13].

## Conclusions

Our two-sample MR approach indicated the causal effect of higher serum TG levels on a higher risk of COVID-19 severity in the European population using the most recent and largest GWAS datasets to date, suggesting that hypertriglyceridemia is a risk factor for COVID-19 severity. However, as the underlying mechanisms remain unclear and our MR study might be biased due to possible horizontal pleiotropy, further studies are warranted to validate our MR findings and investigate underlying mechanisms.

## Supplementary Information

**Additional file 1. Table S1**. Statistical power for estimating effects of genetically predicted lipid-lipoprotein traits on COVID-19 risk in MR analyses.** Table S2**. The characteristics of all SNVs included in MR analyses estimating the causal effect of genetically predicted serum apolipoprotein B levels (exposure) on risk of COVID-19 susceptibility (outcome1), hospitalization (outcome2), and severity (outcome3).** Table S3**. The characteristics of all SNVs included in MR analyses estimating the causal effect of genetically predicted serum LDL-cholesterol levels (exposure) on risk of COVID-19 susceptibility (outcome1), hospitalization (outcome2), and severity (outcome3).** Table S4**. The characteristics of all SNVs included in MR analyses estimating the causal effect of genetically predicted serum triglyceride levels (exposure) on risk of COVID-19 susceptibility (outcome1), hospitalization (outcome2), and severity (outcome3).

**Additional file 2.** **Figure S1**. Scatter plots for estimating causal effects of genetically predicted serum Apo-B levels on risk of (a) susceptibility, (b) hospitalization, and (c) severity.** Figure S2**. Scatter plots for estimating causal effects of genetically predicted serum LDL-C levels on risk of (a) susceptibility, (b) hospitalization, and (c) severity.** Figure S3**. (a) Leave-one-out sensitivity analysis for estimating causal effect of genetically predicted serum TG levels on risk of COVID-19 severity. (b) Funnel plot for estimating causal effect of serum TG levels on risk of COVID-19 severity.

**Additional file 3. Table S5**. Univariable MR results of the effect of risk of COVID-19 on atherogenic lipid-related traits.

## Data Availability

The exposure GWAS dataset is publicly available from the IEU open GWAS database (https://gwas.mrcieu.ac.uk/datasets/) as GWAS-IDs of “ieu-b-108” for serum Apo-B level, “ieu-b-110” for serum LDL-C level, “ieu-b-111” for serum TG level, and “ieu-b-40” for BMI. The exposure GWAS dataset is publicly available from COVID-19-HGI GWAS meta-analyses (Round 5) (https://www.covid19hg.org/results/r5/). The functions used in the TwoSampleMR package are available from the website https://mrcieu.github.io/TwoSampleMR/reference/index.html.

## Abbreviations

Apo-B: Apolipoprotein B
BMI: Body mass index
CAD: Coronary artery disease
CI: Confidence interval
COVID-19: Coronavirus disease 2019
COVID-19-HGI: COVID-19 Host Genetics Initiative
CRP: C-reactive protein
GLGC: Global Lipids Genetic Consortium
GWAS: Genome-wide association study
IgG: Immunoglobulin G
IV: Instrumental variable
IVW: Inverse variance weighted
LDL-C: Low-density lipoprotein cholesterol
MAF: Minor allele frequency
MR: Mendelian randomization
MR-PRESSO: MR-Pleiotropy RESidual Sum and Outlier
OR: Odds ratio
SARS-CoV-2: Severe acute respiratory syndrome coronavirus 2
SD: Standard deviation
SE: Standard error
SNP: Single-nucleotide polymorphism
SNV: Single-nucleotide variant
TG: Triglyceride
UKBB: UK Biobank
