# Supplementary material for: Estimating causal effects of atherogenic lipid-related traits on COVID-19 susceptibility and severity using a two-sample Mendelian randomization approach
Source: BMC Med Genomics. 2021 Nov 13;14:269. doi: 10.1186/s12920-021-01127-2 (PMC8590430; doi:10.1186/s12920-021-01127-2)
Supplement: Supplementary file 2 — Additional file 2. Figure S1. Scatter plots for estimating causal effects of genetically predicted serum Apo-B levels on risk of (a) susceptibility, (b) hospitalization, and (c) severity. Figure S2. Scatter plots for estimating causal effects of genetically predicted serum LDL-C levels on risk of (a) susceptibility, (b) hospitalization, and (c) severity. Figure S3. (a) Leave-one-out sensitivity analysis for estimating causal effect of genetically predicted serum TG levels on risk of COVID-19 severity. (b) Funnel plot for estimating causal effect of serum TG levels on risk of COVID-19 severity. [file 12920_2021_1127_MOESM2_ESM.pptx]

## Slide 1
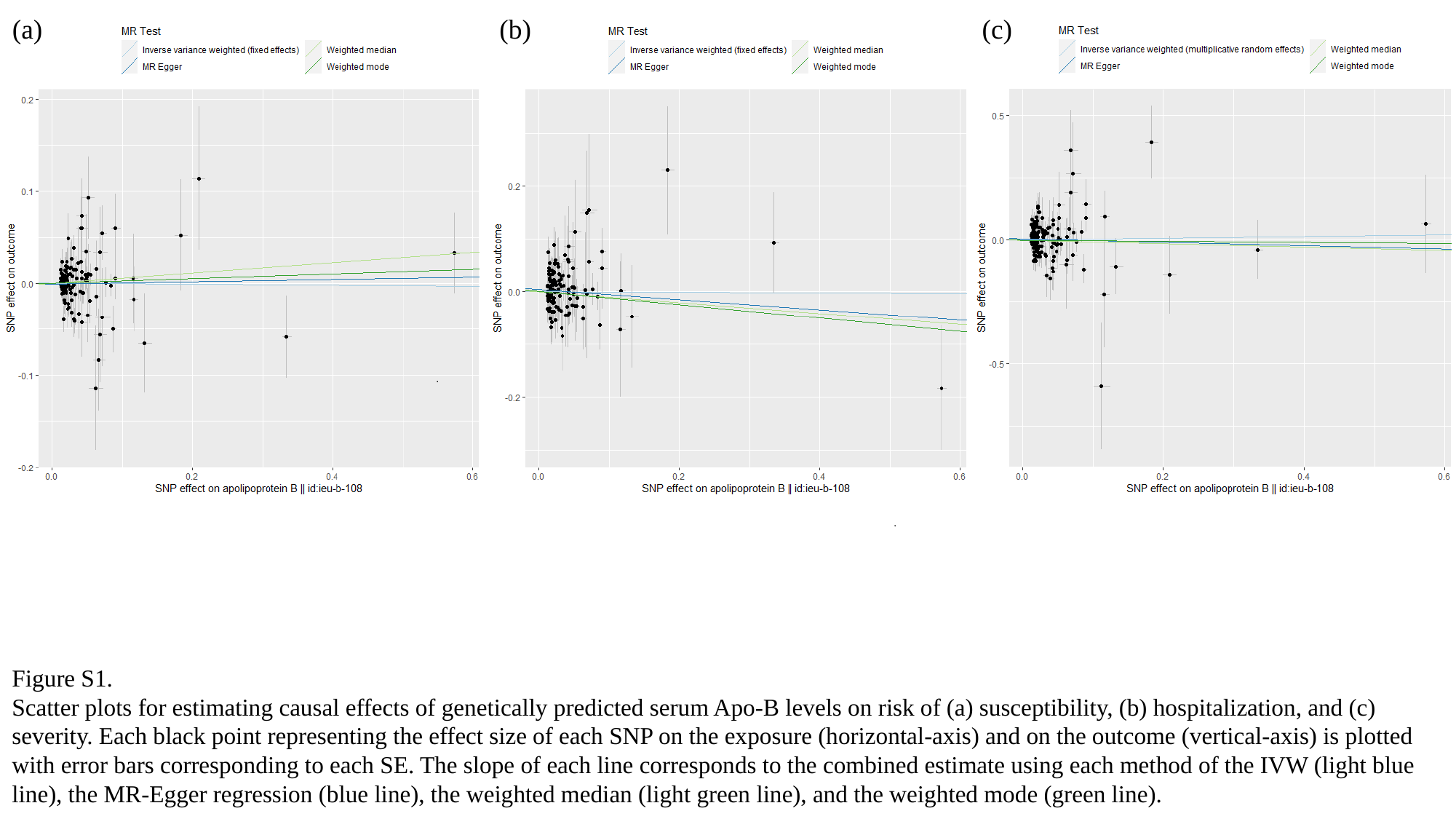

(b)
(c)
(a)
Figure S1.
Scatter plots for estimating causal effects of genetically predicted serum Apo-B levels on risk of (a) susceptibility, (b) hospitalization, and (c) severity. Each black point representing the effect size of each SNP on the exposure (horizontal-axis) and on the outcome (vertical-axis) is plotted with error bars corresponding to each SE. The slope of each line corresponds to the combined estimate using each method of the IVW (light blue line), the MR-Egger regression (blue line), the weighted median (light green line), and the weighted mode (green line).

## Slide 2
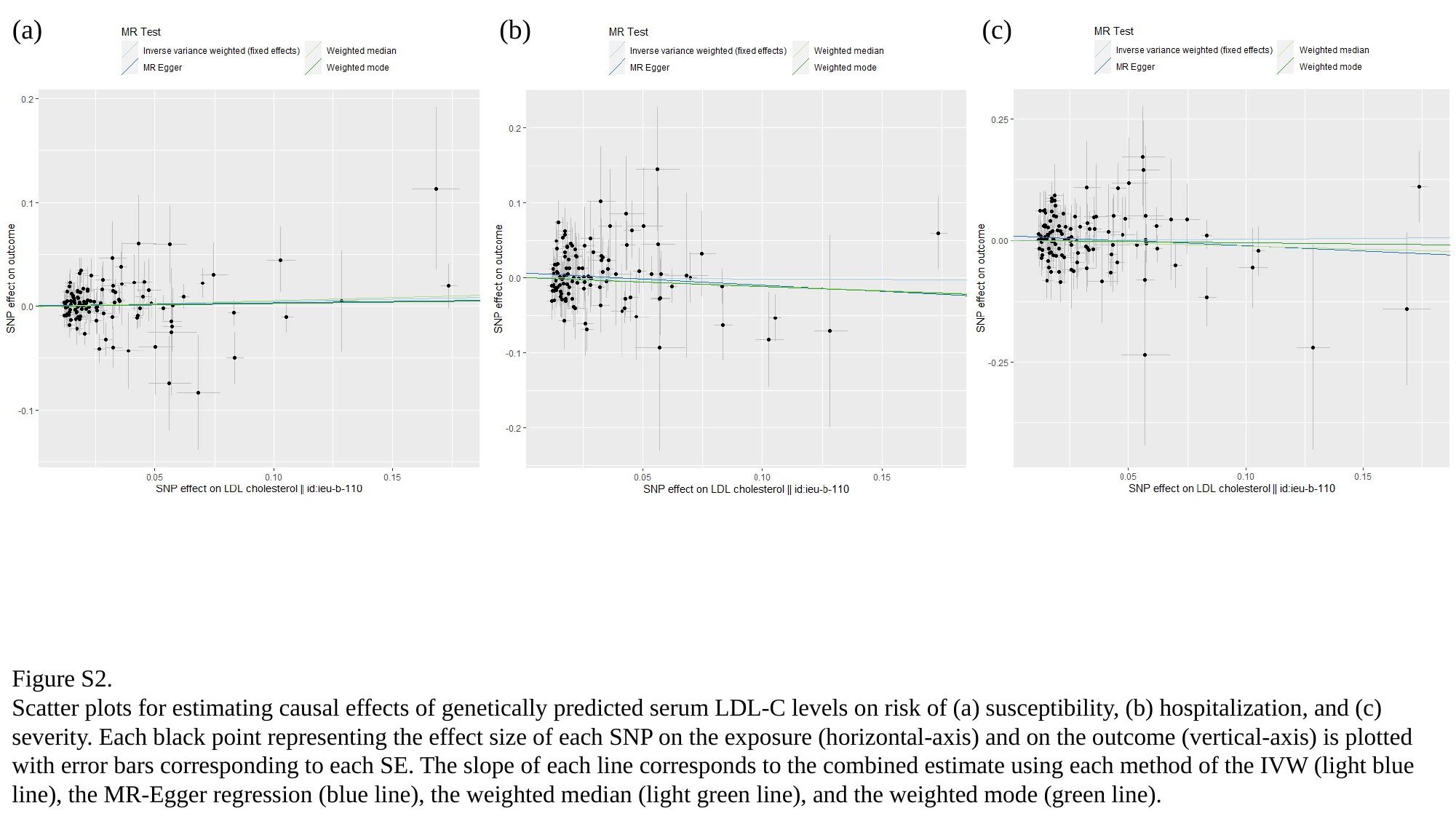

(b)
(c)
(a)
Figure S2.
Scatter plots for estimating causal effects of genetically predicted serum LDL-C levels on risk of (a) susceptibility, (b) hospitalization, and (c) severity. Each black point representing the effect size of each SNP on the exposure (horizontal-axis) and on the outcome (vertical-axis) is plotted with error bars corresponding to each SE. The slope of each line corresponds to the combined estimate using each method of the IVW (light blue line), the MR-Egger regression (blue line), the weighted median (light green line), and the weighted mode (green line).

## Slide 3
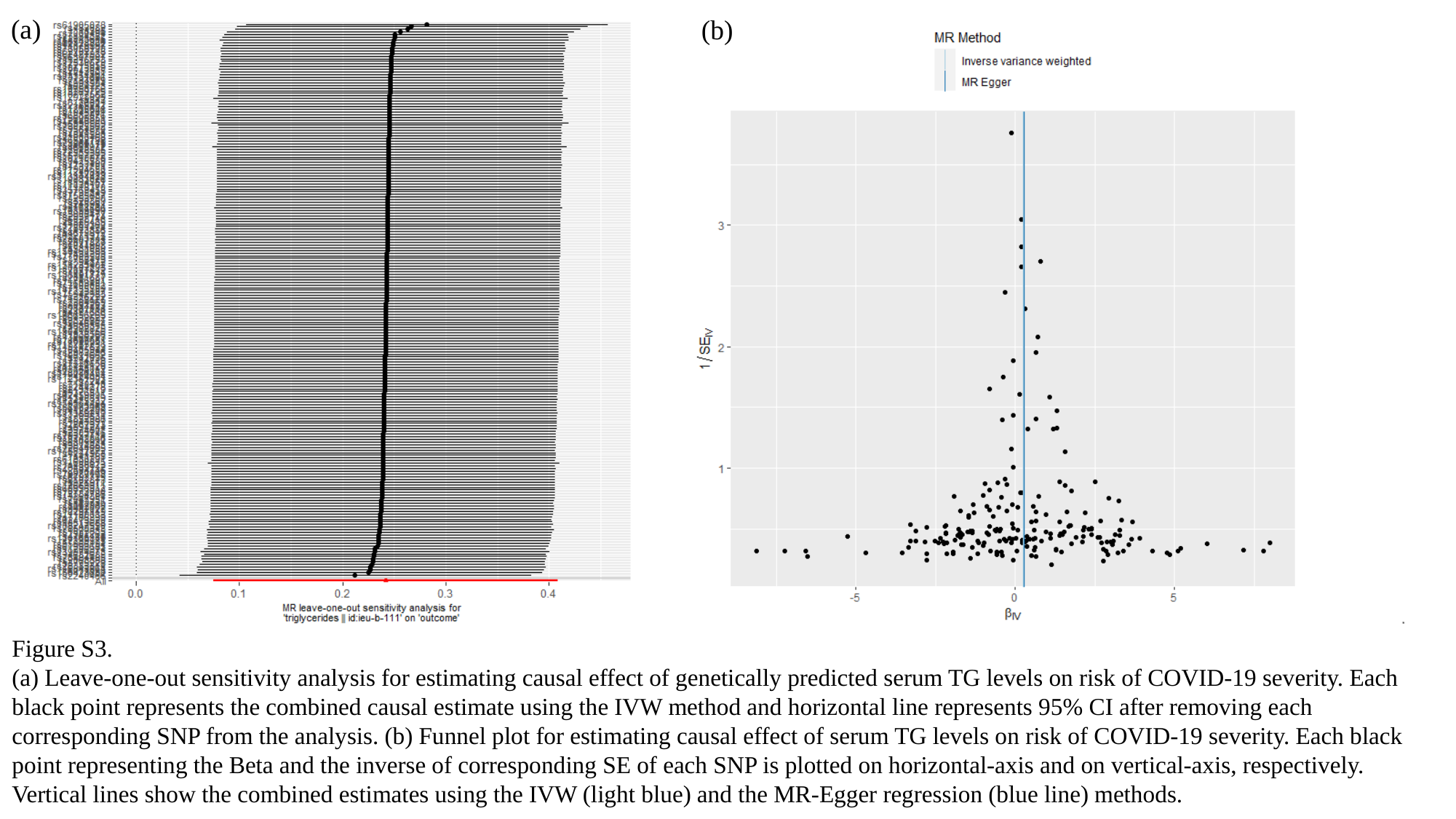

(a)
(b)
Figure S3.
(a) Leave-one-out sensitivity analysis for estimating causal effect of genetically predicted serum TG levels on risk of COVID-19 severity. Each black point represents the combined causal estimate using the IVW method and horizontal line represents 95% CI after removing each corresponding SNP from the analysis. (b) Funnel plot for estimating causal effect of serum TG levels on risk of COVID-19 severity. Each black point representing the Beta and the inverse of corresponding SE of each SNP is plotted on horizontal-axis and on vertical-axis, respectively. Vertical lines show the combined estimates using the IVW (light blue) and the MR-Egger regression (blue line) methods.
