# Supplementary material for: Estimating causal effects of atherogenic lipid-related traits on COVID-19 susceptibility and severity using a two-sample Mendelian randomization approach
Source: BMC Med Genomics. 2021 Nov 13;14:269. doi: 10.1186/s12920-021-01127-2 (PMC8590430; doi:10.1186/s12920-021-01127-2)
Supplement: Supplementary file 3 — Additional file 3. Table S5. Univariable MR results of the effect of risk of COVID-19 on atherogenic lipid-related traits. [file 12920_2021_1127_MOESM3_ESM.docx]

**Table S5.**

**Univariable MR results of the effect of risk of COVID-19 on atherogenic lipid-related traits.**

| Exposure  traits | Outcome  traits | N of  SNVs | IVW  method | MR-Egger regression  method | | Weighted median  method | Weighted mode  method | Heterogeneity  (IVW method) |
| --- | --- | --- | --- | --- | --- | --- | --- | --- |
|  |  |  | Beta (SE)  *P*-value | Beta (SE)  *P*-value | Intercept (SE)  *P*-value | Beta (SE)  *P*-value | Beta (SE)  *P*-value | Cochran’s Q  *P*-value |
| COVID-19  susceptibility | Apo-B | 4 | 0.0870 (0.1021)  0.39 | -0.0437 (0.3218)  0.90 | 0.0131 (0.0300)  0.70 | -0.0288 (0.0198)  0.14 | -0.0238 (0.0178)  0.27 | 172.2  < 0.001 |
| COVID-19  hospitalization | Apo-B | 6 | 0.0007 (0.0162)  0.97 | -0.0174 (0.0422)  0.70 | 0.0046 (0.0097)  0.66 | -0.0105 (0.0060)  0.08 | -0.0126 (0.0064)  0.11 | 57.5  < 0.001 |
| COVID-19  severity | Apo-B | 7 | 0.0047 (0.0114)  0.68 | -0.0021 (0.0319)  0.95 | 0.0022 (0.0093)  0.83 | -0.0077 (0.0051)  0.13 | -0.0080 (0.0050)  0.16 | 55.4  < 0.001 |
| COVID-19  susceptibility | LDL-C | 4 | 0.1218 (0.1350)  0.37 | -0.0244 (0.4310)  0.96 | 0.0147 (0.0402)  0.75 | -0.0161 (0.0191)  0.40 | -0.0178 (0.0175)  0.38 | 298.4  < 0.001 |
| COVID-19  hospitalization | LDL-C | 6 | 0.0035 (0.0167)  0.83 | -0.0129 (0.0437)  0.78 | 0.0041 (0.0100)  0.70 | -0.0094 (0.0059)  0.11 | -0.0098 (0.0057)  0.15 | 60.4  < 0.001 |
| COVID-19  severity | LDL-C | 7 | 0.0063 (0.0118)  0.59 | 0.0011 (0.0329)  0.98 | 0.0017 (0.0096)  0.87 | -0.0062 (0.0053)  0.24 | -0.0069 (0.0050)  0.22 | 58.4  < 0.001 |
| COVID-19  susceptibility | TG | 4 | -0.0457 (0.0530)  0.39 | -0.0533 (0.1747)  0.79 | 0.0008 (0.0163)  0.97 | -0.0071 (0.0170)  0.68 | -0.0065 (0.0171)  0.73 | 50.6  < 0.001 |
| COVID-19  hospitalization | TG | 6 | 0.0033 (0.0120)  0.78 | -0.0136 (0.0308)  0.68 | 0.0043 (0.0071)  0.58 | -0.0031 (0.0058)  0.59 | -0.0056 (0.0058)  0.37 | 34.5  < 0.001 |
| COVID-19  severity | TG | 7 | 0.0046 (0.0086)  0.59 | 0.00043 (0.0240)  0.99 | 0.0013 (0.0070)  0.86 | -0.0023 (0.0047)  0.63 | -0.0034 (0.0053)  0.55 | 34.2  < 0.001 |

**Abbreviations:** Apo-B, apolipoprotein B; COVID-19, coronavirus disease 2019; IVW, inverse variance weighted; LDL-C, low-density lipoprotein cholesterol; MR, Mendelian randomization; N, number; SE, standard error; SNV, single nucleotide variant. TG, triglyceride.
